# **Long-term random sampling confirms high-use areas and indicates declining abundance of juvenile smalltooth sawfish (** ***Pristis pectinata*** **) in Charlotte Harbor, Florida**

**DOI:** 10.1038/s41598-025-14430-0

**Published:** 2026-03-10

**Authors:** Nicholas A. Farmer, Adam B. Brame, Rabiya Dar, Andrew K. Wooley, Lukas B. Heath, Dylan M. Yakich, Steven M. Lombardo, Gregg R. Poulakis

**Affiliations:** 1 NOAA/National Marine Fisheries Service, Southeast Regional Office, 263 13th Avenue South, St. Petersburg, FL 33701 USA; 2 NOAA/National Centers for Coastal Ocean Science, 1305 East West Highway, Silver Spring, MD 20910 USA; 3 Charlotte Harbor Field Laboratory, Fish and Wildlife Research Institute, Florida Fish and Wildlife Conservation Commission, 585 Prineville Street, Port Charlotte, FL 33954 USA; 4 Bonefish and Tarpon Trust, 2937 SW 27th Avenue, Suite 203, Miami, FL 33133 USA

**Keywords:** Charlotte Harbor, Endangered species, Population size, Recovery, Relative abundance, Smalltooth sawfish, Species distribution model, Ocean sciences, Marine biology

## Abstract

We evaluated 13 years of fishery-independent small juvenile (< 1.86 m stretch total length) endangered smalltooth sawfish sampling data from southwest Florida’s Charlotte Harbor estuarine system. From 2010 through 2022, within the sampling domain, estimated abundance declined, with an annual average across all seasons of 34 ± 11 individuals (range: 15–69). High-use areas were spatiotemporally stable and associated with well-oxygenated (dissolved oxygen > 7 mg/L), warm (25–33 °C), and brackish (salinity 5–27) waters between 5 and 15 km upriver along natural shorelines with concentrations of mangroves in both riverine nurseries. Adult female abundance, back-calculated from expected brood size (48 ± 16, range: 26–144) and stable age distribution (87 ± 30, range: 39–211), was low. Our findings of a significant declining trend in relative abundance of small juveniles and limited numbers of adult females are concerning considering ongoing threats from commercial shrimp trawl bycatch, destruction of nursery habitats, and major recent mortality events. Our model facilitates estimation of the number of individuals potentially exposed to harmful anthropogenic activities and identifies perennial high-use areas as priorities for permanent protection from habitat alterations and loss to promote recovery of the endangered smalltooth sawfish population in this unique estuarine system.

**Supplementary Information:**

The online version contains supplementary material available at 10.1038/s41598-025-14430-0.

## Introduction

Understanding spatial and temporal trends in species abundance is essential for effective management and conservation. The spatiotemporal abundance of marine species is influenced by many abiotic and biotic factors [1]. Models accounting for these drivers provide opportunities for prediction from sample locations to the scale of a sample domain [2]. They also provide spatiotemporally explicit estimates of abundance and uncertainty, even for populations where individuals are not uniformly distributed over the study area [3]. This information can be used for monitoring population trends [4], marine spatial planning [5–7], and other management practices [8, 9]. Quantifying habitat requirements and population abundance is particularly challenging for endangered species where encounters in traditional abundance surveys are rare.

Sawfishes are among the world’s most endangered marine fishes and are associated with coastal marine waters, estuaries, and rivers [10]. They are benthic rays with a distinct rostrum—a long, flat snout edged with teeth—often referred to as a saw. Globally, all five species are threatened with extinction because of low intrinsic rates of population increase, susceptibility to bycatch in fisheries owing to the easy entanglement of their rostra in a variety of gears, and habitat loss [11]. In the United States (U.S.), the smalltooth sawfish (*Pristis pectinata*) was distributed from North Carolina to Texas, but was always most abundant in south Florida [12]. Due primarily to fisheries bycatch, there was a marked decline in the entire Atlantic smalltooth sawfish population in the twentieth century that left it a fraction of its historical size and distribution [13, 14]. Currently, viable populations only exist in the southeastern U.S. [10], and The Bahamas [15], with a few recent reports from Mexico [16] and Cuba [17]. In 2003, the U.S. distinct population segment was listed as endangered under the Endangered Species Act (ESA) [18].

Currently, there is a resident, reproducing population of smalltooth sawfish in south and southwest Florida [10]. In Charlotte Harbor, recruitment occurs between November and July, peaking between April and May [19]. The species undergoes ontogenetic habitat shifts [20–23], with small juveniles (< 2 m stretch total length, STL) generally restricted to shallow estuarine waters (< 1 m deep, [19]) with mud or sand bottoms [20, 24] near red mangrove shorelines [25]. The shallow, mangrove-lined waters provide refuge from predators and an abundant supply of their fish prey [26, 27]. As they grow to age 2–3 (at approximately 2.2 m STL), they leave the shallows for a broader array of habitats [10].

Adult females are philopatric, typically returning to the same nursery to give birth every other year [28]. Tracking studies in multiple nurseries have shown that small juveniles show high site fidelity to parturition sites [29–31]. In the U.S., the main threats to small juveniles are habitat modification such as removal of red mangroves and loss of shallow habitats because of coastal development, entanglement in marine debris, and poor handling associated with recreational fishing, including intentional, illegal removal of their rostrums [32–34]. Migratory large juveniles and adults face similar threats along with bycatch in coastal fisheries, particularly the largely unmonitored southeast shrimp trawl fishery [23, 35, 36].

In 2009, the U.S. National Marine Fisheries Service (NMFS) designated two critical habitat units for juvenile smalltooth sawfish, one in the Charlotte Harbor estuary and one in Everglades/Ten Thousand Islands [26]. These areas contain the only known remaining nursery habitats for the U.S. DPS, based on criteria for elasmobranchs [37]: (1) juveniles are more commonly encountered than in other areas; (2) juveniles remain or return for extended periods; and (3) the area is repeatedly used by juveniles across years.

In this study, we used fishery-independent gillnet survey data collected in the Charlotte Harbor estuarine system during 2010–2022 to characterize the spatial distribution, relative abundance, and the abundance trend of age-0 and age-1 smalltooth sawfish. Specifically, the goals of this study were to: (1) develop spatiotemporally explicit models of juvenile density, (2) identify key environmental features that predict the presence of juveniles, (3) provide an estimate of population abundance for juveniles, (4) evaluate temporal trends in relative abundance of juveniles, and (5) back-calculate estimates of adult females contributing to the estuarine population. Ultimately, a better understanding of the abundance trend for small juvenile smalltooth sawfish and the number of reproducing females contributing to this juvenile population will enhance our understanding of the population’s capacity to recover in the context of current estimated levels of mortality, both natural and anthropogenic.

## Methods

### Study area

**Fig. 1:**
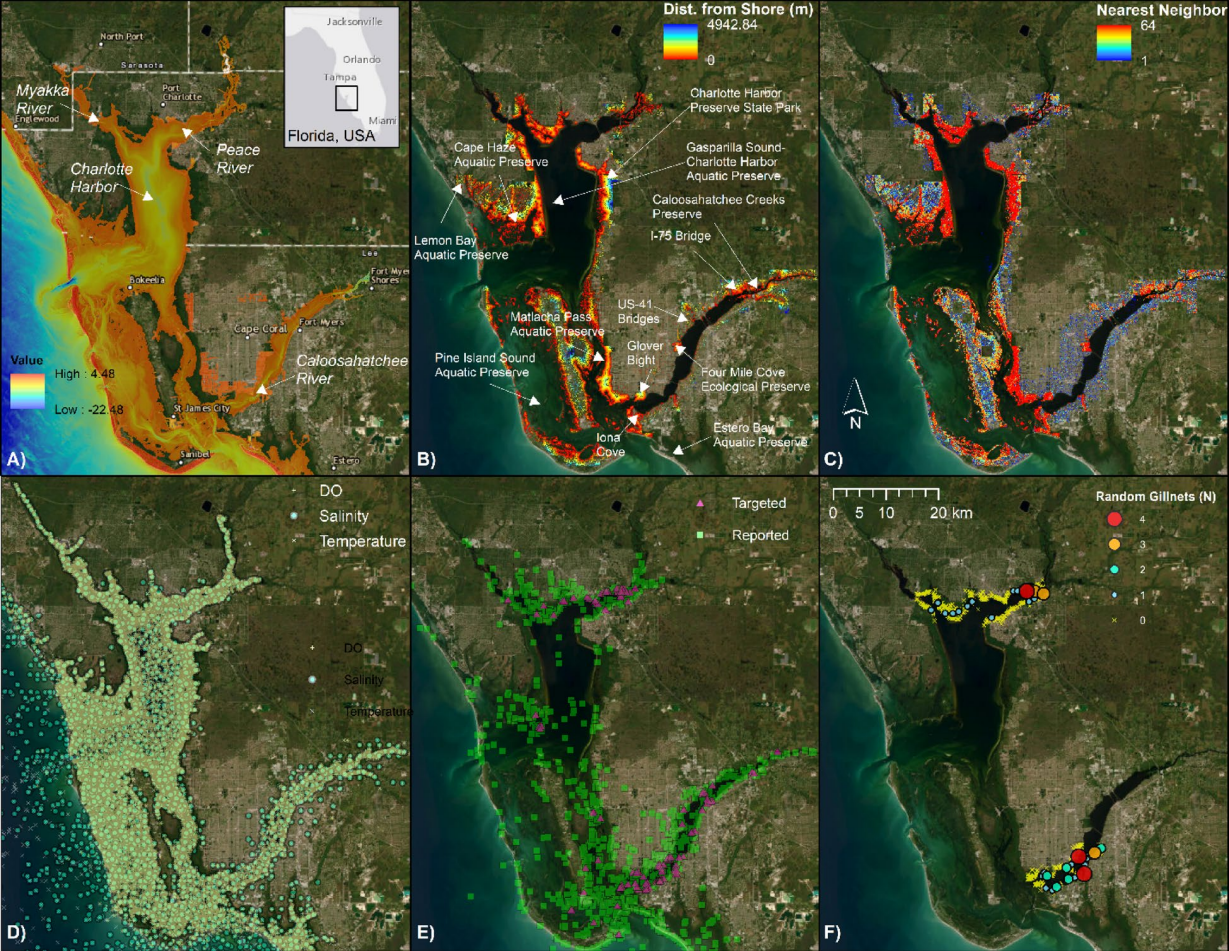
Charlotte Harbor study area and data layers. (**A**) Bathymetry (m) and locations of the Myakka, Peace, and Caloosahatchee Rivers; (**B**) Mangrove distance from shoreline and locations of preserves; (**C**) Mangrove nearest neighbor analysis identifying clusters of mangroves; (**D**) Daily sonde data for temperature (°C), dissolved oxygen (DO), and salinity used for interpolation; (**E**) Smalltooth sawfish (*Pristis pectinata*) catch locations (zeroes excluded) from targeted sampling by the Florida Fish and Wildlife Conservation Commission (age-0 and age-1 only) and the U.S. Sawfish Recovery Database (all life history stages); and (**F**) Sampling and catch locations from random gillnet sampling. Basemap used with permission from ESRI World Imagery and its partners.

At approximately 700 km^2^, the Charlotte Harbor estuarine system is one of the largest estuaries in Florida and includes six aquatic preserves covering over 75,000 ha of water and fringing mangroves (Fig. 1A and B). The northern portion of the estuarine system is fed by the Peace River from the northeast and Myakka River from the northwest. Compared to the Caloosahatchee River that feeds the southern portion of the Harbor, the Peace and Myakka Rivers have relatively unaltered freshwater flow regimes. The northern portion of the system between the two rivers is characterized by natural, mangrove-lined shorelines (Fig. 1C).

The mouth of the Peace River, surrounded by the cities of Port Charlotte to the north and Punta Gorda to the south, is heavily developed, and contains complex networks of human-made waterways. East of the I-75 bridge, there are some large patches of natural mangrove habitat along the shoreline and along the perimeter of several mid-river islands (e.g., Bird Key, Long Island). Further upriver, the lower Peace River extends to the north and Shell Creek extends to the east. Both contain long stretches of natural shoreline and shallow creeks running between mangrove islands.

The southern portion of the estuarine system contains the Caloosahatchee River, a highly altered, flow-managed river characterized by extensive canal systems and water control structures used by the South Florida Water Management District to regulate overall river flow and manage water levels in Lake Okeechobee. The region contains the cities of Cape Coral and Fort Myers, and features a complex network of human-made waterways with myriad hardened shorelines and substantial coastal development. Stretches of red mangrove shoreline in the Caloosahatchee River are limited mostly to mid-river islands (e.g., Ward Island, Marsh Point, Midway Island, Beautiful Island), the northeast side of the US-41 bridges, Four Mile Cove Ecological Preserve (1.4 km^2^), and the Caloosahatchee Creeks Preserve (5.3 km^2^; Fig. 1B). The only remaining red mangrove shorelines in the lower river are in Glover Bight and Iona Cove.

### Mangrove classification

NMFS designated juvenile smalltooth sawfish critical habitat under the U.S. Endangered Species Act and identified the habitat features essential to the conservation of the species (also known as the “essential features”) as “…red mangroves and shallow euryhaline habitats characterized by water depths between the Mean High-Water line and 3 ft (0.9 m) measured at Mean Lower Low Water (MLLW)” [26].

To identify mangrove habitats in the estuarine system, National Agriculture Imagery Program (NAIP) multi-spectral (4-band) imagery, at 0.6-m resolution, was mosaicked within ArcGIS (ESRI, Redlands, CA). Mangroves were not categorized by species due to resolution limitations; however, most shoreline mangroves were likely red mangroves. Supervised classification was conducted in ArcGIS to determine whether imagery pixels contained mangroves and involved three steps: (1) collect training samples, (2) create a signature file, and (3) run a maximum likelihood classifier. Training samples consisted of 90 points randomly chosen from among “mangrove,” “other vegetation,” and “non-vegetation” ground truth points within 30 m of the shoreline (30 for each class) in the northern estuary near the Peace River nursery and an additional 90 points from the southern estuary near the Caloosahatchee River nursery. Next, the ‘Create Signatures’ tool was used to take information from the training samples to produce an ASCII signature file with information on the classes, including how many cells were classified under that class and covariances across all raster bands (e.g., RGB, NIR). Finally, the ‘Maximum Likelihood Classification’ tool was run with a priori probability weighting, with the probability of being classified as a mangrove reduced to 2% in urban regions. Urban regions were identified using the Florida Department of Environmental Protection’s (FDEP) Statewide Land Use Land Cover (LULC) “Urban and Built Up” data (2012–2019). This step reduced the probability of lawns and landscape vegetation being classified as mangroves. The probability of “other vegetation” and “non-vegetation” were kept equal and totaled 98% in these urban regions. A priori weighting was kept equal between all three classes for areas within the study extent not overlapping with the urban dataset. This required two runs of the ‘Maximum Likelihood Classification’ tool: once for urban areas and again for non-urban areas. After finalizing the classification, ArcGIS’s ‘Set Null’ tool was used to set all pixels not classified as mangrove to null.

Because small juvenile sawfish can use only submerged edges of mangroves as cover from predation, the distance to shoreline was calculated for all cells classified as mangrove within the study extent. This was accomplished in ArcGIS, using the ‘Euclidean Distance’ tool by measuring from the shoreline layer to the center of mangrove raster cells within a buffer 10 m from the shoreline (Fig. 1B).

To determine the relative quality of surrounding habitat for each mangrove cell, a nearest neighbor analysis was performed. Using the ‘Focal Statistics’ tool, all mangrove cells were summed within an 8 × 8 cell neighborhood (23.04 m^2^) for each classified cell. The result was a raster of cells with values from 1 to 64 where 1 indicates that the only mangrove within the neighborhood is the one at the center and 64 indicates that all cells contained mangroves (i.e., continuous mangrove coverage) (Fig. 1C).

### Field sampling

Data were obtained from a variety of sources. Daily and sub-daily environmental data have been collected by sondes and field staff during the Florida Fish and Wildlife Conservation Commission (FWC) fisheries-independent monitoring (FIM) program sampling activities for several decades (Fig. 1D). Similarly, since the late 1990s, sawfish encounters by the public have been reported to the U.S. Sawfish Recovery Hotline [e.g., 24, 38, 39] and, from 2004 to present, FIM has conducted targeted sampling for sawfish based largely on sightings reports (Fig. 1E). In addition, random gill net sampling has occurred since 2010 within 200-m square “micro grids” pre-selected from within 1 × 1 cartographic grids (i.e., 1 square nautical mile) containing water depths up to 3 m (see [40] for details). Briefly, two random trips per month were conducted in northern Charlotte Harbor (eight sites per month; two nets per site) and the lower Caloosahatchee River (six sites per month; two nets per site). Two 30.5-m (100 ft) or two 61-m (200 ft) gill nets with 102 mm (4 in) stretch monofilament mesh were set perpendicular to shore approximately 100 m apart. In general, the 61-m nets were used unless sampling was restricted to a confined space (e.g., a small creek or canal). Nets were soaked for 1 h and checked after 30 min or whenever fishes of any species were caught (e.g., when splashing was observed). Locations and STL of sawfish were recorded (Fig. 1F; Figs. S1–3). Protocols were approved by NOAA Fisheries Office of Protected Resources and Florida Fish and Wildlife Research Institute, and conducted under the authority of NOAA Fisheries Endangered Species Act 10(a)(1)(A) permit numbers 15802 and 21043 (FWC).

### Spatial abundance modeling

We fit generalized additive models (GAMs) to all possible permutations of spatially- and temporally-varying environmental covariates that could correlate with sawfish distributions, including physiographic, physical oceanographic, and biological covariates, using the *mgcv* package in R (Table 1, [41, 42]. We accounted for non-uniform sampling effort by including log-transformed effort (i.e., soak area) as an offset [43, 44]. We selected a GAM approach over a generalized linear modeling approach because of the flexibility of GAMs and the anticipated nonlinear relationships between abundance and covariates [45].

**Table 1:** Covariates considered in density surface modeling.

Covariate	Description
Min_Depth	Minimum depth (m) in micro-grid, determined from BlueTopo bathymetry
Avg_DO	Mean dissolved oxygen (mg/L) value across samples on given survey date within a micro-grid
Avg_temp	Mean temperature (°C) across samples on given survey date within a micro-grid
Avg_salin	Mean salinity across samples on given survey date within a micro-grid
Avg_pH	Mean pH across samples on given survey date within a micro-grid
Min_MDTS	Minimum mangrove distance to shore (m)
Sum_MNN	Sum of mangrove nearest neighbor values from Zonal Statistics
Year	Year of sampling
Season	“Spring/Summer” (March–August) or “Fall/Winter,” based on month of sampling
River	River nearest sampling location (i.e., Caloosahatchee, Myakka, or Peace)
DistToRM	Distance from sampling location to nearest river mouth (m), with locations outside of river expressed as negative distances
DevelopedSL	Sampling grid exclusively contains developed shoreline (1) or contains some natural shoreline (0)

Preparing covariates for spatial abundance model fitting was performed in ArcGIS 10.8, using data projected in UTM NAD 1983 Zone 17N. Bathymetry data for the lower Myakka, Peace, and Caloosahatchee Rivers and Charlotte Harbor were downloaded from the NOAA Office of Coast Survey’s BlueTopo curated collection of high-resolution seafloor models (nowCoast: https://nowcoast.noaa.gov/, accessed October 2023). All layers were merged using ArcGIS’s ‘Mosaic to New Raster’ function (Fig. 1A). Water depth was added to random gillnet survey point data using ArcGIS’s ‘Add Surface Information’ tool. Point data were assigned to FWC sampling microgrids using a ‘Spatial Join’ function. Bathymetric characteristics, including minimum, maximum, and mean depth and slope, were assigned to microgrids using the ‘Add Surface Information’ function. Similarly, mangrove classification information was computed for each microgrid using the ‘Zonal Statistics as Table’ function. A ‘Join’ function was used to assign all statistics for mangrove distance to shore and mangrove nearest neighbor to microgrids. Sampling microgrids containing exclusively developed shorelines were visually identified from aerial imagery from ESRI.

Mangrove statistics were imported into R and joined to microgrids using package *dplyr*’s ‘left_join’ function. Sampling effort (i.e., soak area) was expressed as yard-minutes, computed as the difference between start and end time in minutes multiplied by net area (in square yards). The distance of each sample point from the river mouth was calculated in ArcGIS using ‘Proximity Tools’. A factor variable ‘River’ was defined by the closest river mouth to the point. A factor variable ‘Season’ was defined as “Spring/Summer” for March–August and “Fall/Winter” for the remaining months. Sawfish catches were restricted to individuals of 1.86 m STL or less, approximating age-0 and age-1 year classes [46].

GAMs were fit across 1024 permutations of the final model covariates (Table 1) using a Tweedie distribution [2] with automatic selection of the power parameter and restricted maximum likelihood (REML, [42]), appropriate for zero-inflated, overdispersed abundance data [47]. To minimize effects of collinearity, correlated predictor variables (i.e., ρ > 0.7) were not included in the same model. Similarly, predictive variables that could not be extrapolated to the broader sampling domain were not included. For example, preliminary analysis suggested that juveniles were caught more frequently on mud bottom; however, benthic substrate characterization beyond the sampling area was not available.

A suite of best-fitting models were identified by Akaike’s Information Criterion (AIC, [48]). All models within two AIC points were tested for predictive utility using internal tenfold cross-validation. Cross-validation was performed using the *caret* [49] and *pROC* packages in R [50] to compute root mean square error (RMSE) and area under the curve (AUC), with the median predicted value for the training dataset used as the pseudo-binary classification threshold for construction of the receiver operating curve (ROC). The best-performing model was selected based on the highest AUC.

We used an external validation metric to gauge consistency between model predictions and the locations of independent sightings of smalltooth sawfish [51]. We divided predictions from the best-fitting abundance model for point-specific independent sightings by domain-wide daily median Z-score transformed predictions across valid water depths (0–3 m), then centered these transformed scores to zero by subtracting one. The greater the proportion of retained Z-scores above zero, the higher the consistency between model predictions and independent observations [51–53].

### Relative abundance estimation

To estimate population relative abundance by extrapolating model fits to the broader sampling domain, a predictive grid was generated by limiting the FWC sampling microgrids to within 61 m of the shoreline and removing areas with water depths > 3 m (i.e., the maximum depth of the gillnet). Soak area was computed as the area for each polygon. Cells were identified as within the sampling universe based on the presence of a FIM set within the cell over the time series. Predictions were made both within the sampling universe and across all valid nearshore cells in the Charlotte Harbor estuarine system.

Daily and sub-daily water temperature, dissolved oxygen (DO), and salinity data were obtained from the FWC’s FIM database independent of project code, U.S. Environmental Protection Agency Water Quality Data [54], South Florida Water Management District dbHydro/dbInsight DBHYDRO [55], and NOAA/FWC Harmful Algal Blooms Observing System [56]. This meta-analysis from 2010 to 2023 generated 187,380 temperature records across 8700 sampling days, 148,877 dissolved oxygen records across 6608 sampling days, and 165,449 salinity records across 7749 sampling days (Fig. 1D). Sub-daily sonde data, including vertical profiles taken during FIM sampling, were averaged at each sampling location by day, yielding 115,913 daily temperature records across 54,514 unique sampling locations, 97,371 daily dissolved oxygen records across 49,565 unique sampling locations, and 103,795 daily salinity records across 52,150 unique sampling locations. Daily spatial environmental data were interpolated across the Charlotte Harbor domain by year and season using the ‘Empirical Bayesian Kriging’ function in ArcGIS with a custom Python script (Figs. S4–S6).

Relative abundance of age-0 and age-1 smalltooth sawfish was estimated within the gillnet sampling domain and the broader Charlotte Harbor estuarine system, following [57]. Using the ‘gam.mh’ function in the *mgcv* package in R [41], the posterior distribution of the GAM parameters was sampled 1000 times to generate a distribution of model coefficients that reflect the statistical uncertainty in the parameter estimation. Predictions of smalltooth sawfish density were generated for each year in the 2010–2022 period based on each of these 1000 parameter sets, capturing both inter-annual variability in environmental conditions and model uncertainty in the resulting samples. The uncertainty from catchability was not propagated throughout the GAM; therefore, the total variance was underestimated; however, based on targeted surveys (e.g., when a sawfish was sighted prior to a set being made), catchability was estimated near 100% (G. R. Poulakis, pers. comm.). Thus, variance in catchability reflects a minor component of the overall uncertainty in density predictions. Extreme values, associated with density predictions orders of magnitude higher than the observed median, reflect projection of the model predictions into poorly sampled parameter space and were excluded prior to variance estimation. The trimmed distribution of density projections was used to summarize predicted average densities within seasons and to calculate metrics of uncertainty. Pairwise comparisons using ANOVA with post-hoc Tukey testing were used to evaluate significance of interannual differences in predicted density. Raw catch per unit effort (CPUE) data were also evaluated for interannual trends in age structure among the captured juveniles, assuming males under 1.34 m and females under 1.38 m were age-0 and any fish above 1.86 m was older than age-1 [58].

The number of adult females required to generate the estimated juvenile abundance was estimated based on: (1) brood size and (2) stable age distribution. In the brood size approach, the estimated total juvenile abundance was divided by the expected brood size of 7–14 individuals [10]. In the stable age distribution approach, 50% (i.e., females only) of the estimated total juvenile abundance was applied to the age-0 and age-1 percentiles in the stable age distribution from [59] to determine the resultant adult female population (e.g., ages 7–11 + , [60]). In this approach, a truncated normal distribution (μ = 251.481, σ = 81.41511, a = 178.511, b = 504.7369) was used to generate 1000 realizations of age-0 and age-1 females and a uniform distribution (a = 7, b = 11) was used to set the lower bound for maturity. Ratios from the stable age distribution were then used to generate 1000 realizations of the resultant number of adult females. In both approaches, the resultant expected number of adult females contributing to offspring in Charlotte Harbor was compared to the estimated number of adult females from parental genotype reconstruction by [28].

## Results

### Mangrove classification

A total of 134 million pixels were identified as mangroves by the supervised classification within the model domain (Fig. 1). Of these, 25 million were in the three rivers, with the majority (50.4%) in the Caloosahatchee River, followed closely by the Peace River (42.2%). The highest concentrations of mangroves in all rivers were around the river mouth, followed by the upper river (Fig. 1B, C). The mid-river stretches of the Peace and Caloosahatchee Rivers were characterized by heavy development with limited patches of concentrated mangrove habitats (Fig. 1C).

### Field sampling

From 2010 through 2022, 2543 random gillnets were set, ranging from 96 in 2010 to 224 in 2011 and capturing a total of 99 juveniles under 1.86 m STL. The number encountered by year ranged from 14 in 2011 to 1 in 2020. Effort averaged 3.7 × 10^6^ ± 0.7 × 10^6^ yard-minutes (range: 2.2 × 10^6^ in 2010 to 4.8 × 10^6^ in 2011 yard-minutes). CPUE averaged 2.1 ± 0.96 sawfish per 10^6^ yard-minutes (range: 0.36 in 2020 to 3.7 in 2010 per 10^6^ yard-minutes).

From 2004 through 2022, 460 targeted gillnets were set, capturing 753 sawfish. Between one and 11 sawfish were captured per set, with a single individual captured on 71% of sets.

Abiotic conditions varied widely at sample locations over the time series. Water temperature ranged from 14.7 to 35.8 °C; sawfish were captured in 18–32.9 °C. Dissolved oxygen ranged from 0.4 to 11.9 mg/L; sawfish were captured in 3.9–11.6 mg/L. Salinity ranged from 0.1 to 35.9; sawfish were captured in 0.2–26.6.

### Spatial abundance modeling

**Fig. 2:**
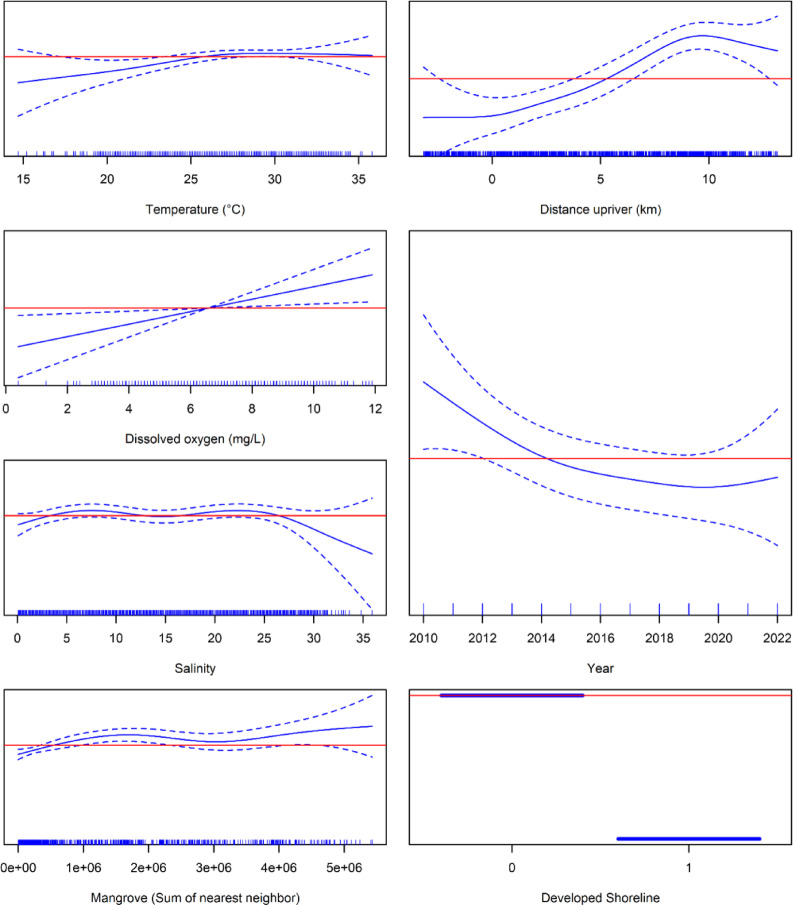
Smalltooth sawfish (*Pristis pectinata*) density model fits. Partial effects plots of relative spline fits for the species abundance model (generalized additive model, GAM) predictor terms for smalltooth sawfish, including water temperature (°C), dissolved oxygen (mg/L), salinity, mangrove density (expressed as sum of nearest neighbor scores), year, distance from river mouth (km), if sampling location was in a grid containing exclusively developed shorelines (0: No, 1: Yes). Solid blue lines denote spline fit; dashed blue lines denote 95% confidence bands. Red lines denote reference levels (0).

**Fig. 3:**
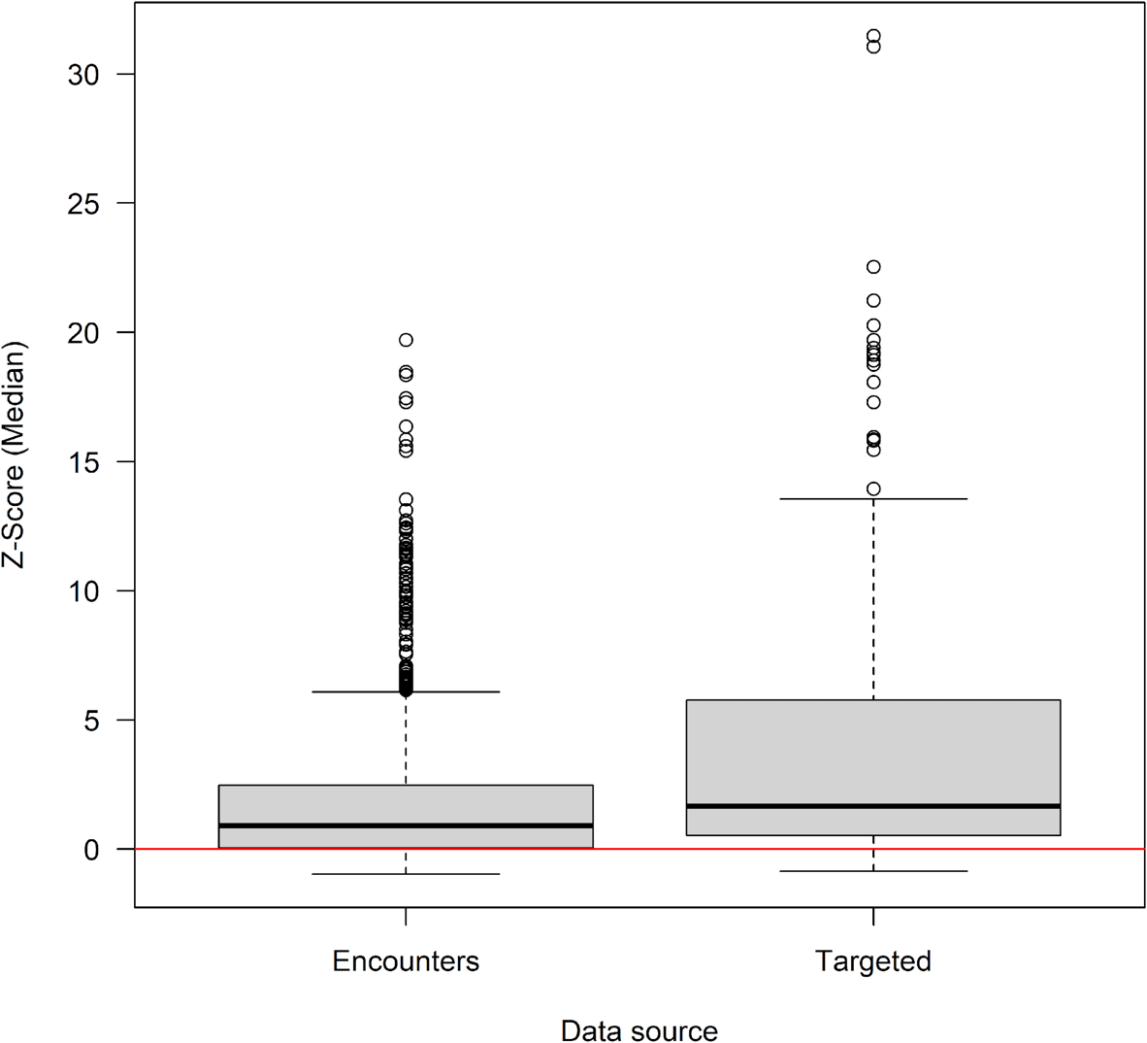
External validation of the smalltooth sawfish (*Pristis pectinata*) distribution model. Predictive utility of best-fitting density model as evidenced by median Z-score standardized probabilities for independent observations of for age-0 and age-1 individuals from targeted sampling from November 2004 through December 2022 using gillnets; and sawfish encounters (“Encounters”) of all life history stages, reported to the U.S. Sawfish Recovery Database (2015–2023). Positive Z-scores (i.e., above red line) indicate consistency between independent observations and model predictions, with higher Z-scores indicative of greater predictive utility.

The best-fitting model explained 14.6% of residual deviance in random gillnet captures (Table 2). Model fits were primarily driven by DO, water temperature, presence of natural shorelines, year, and mangrove concentration, with lesser contributions from salinity and distance to river mouth. The model predicted significantly higher catches near natural shorelines with high concentrations of mangroves in well-oxygenated (DO > 7 mg/L), warm (25–33 °C), brackish (salinity between 5 and 27) waters between 5 and 15 km upriver (Fig. 2). Internal cross-validation of the best-fitting abundance model revealed relatively low predictive error with RMSE = 0.26 ± 0.05 (range: 0.16–0.32), and ‘poor’ to ‘excellent’ predictive ability [61] with AUC = 0.63 ± 0.11 (range: 0.52–0.91). External validation suggested good predictive utility to independent observations, with 78% of predictions above threshold (Fig. 3). The median Z-score standardized probability of observation was significantly greater than 0 [*t*(1973) = 25.939, *p* < 0.0001, (95% CI) = 2.08 (1.92–2.23)]. External validation indicated good predictive utility for targeted gillnet sampling for age-0 and age-1 individuals, with 86% of predictions above threshold [*t*(263) = 11.402, *p* < 0.0001, (95% CI) = 4.55 (3.76–5.33)]. Predictive utility for encounter data representing individuals of all age classes was also good, with 76% of predictions above threshold [*t*(1709) = 26.404, *p* < 0.0001, (95% CI) = 1.69 (1.57–1.82)].

**Table 2:** Model fit summary for best-fitting model, where SE denotes standard error, t denotes t value, F denotes *F* value, Rel.Dev.Exp. denotes relative deviance explained, edf denotes estimated degrees of freedom, Ref.df denotes reference degrees of freedom, and p-value denotes significance.

	Estimate	SE	t	*p*-value	Rel.Dev.Exp
Parametric coefficients					
(Intercept)	− 13.5152	0.1471	− 91.863	< 0.001***	
DevelopedSL1	− 0.1036	0.3941	− 0.263	0.793	19.7%

	edf	Ref.df	F	*p*-value	
Approximate significance of smooth terms					
s(Avg_DO)	1.000	1.000	6.121	0.01343	21.8%
s(Avg_temp)	2.244	2.861	2.539	0.05934	20.9%
s(Avg_salin)	4.177	5.145	1.606	0.15293	8.1%
s(Sum_MNN)	3.422	4.242	4.351	0.00137	13.0%
s(Year)	1.885	2.344	2.434	0.08042	15.5%
s(distToRM)	3.593	4.461	9.890	< 0.001***	1.0%

Significance code: < 0.001 ‘***’.

R^2^(adjusted) = 0.0103.

Deviance explained = 14.6%.

Restricted maximum likelihood = 342.58.

Scale estimate = 1.0421.

n = 2373.

**Fig. 4:**
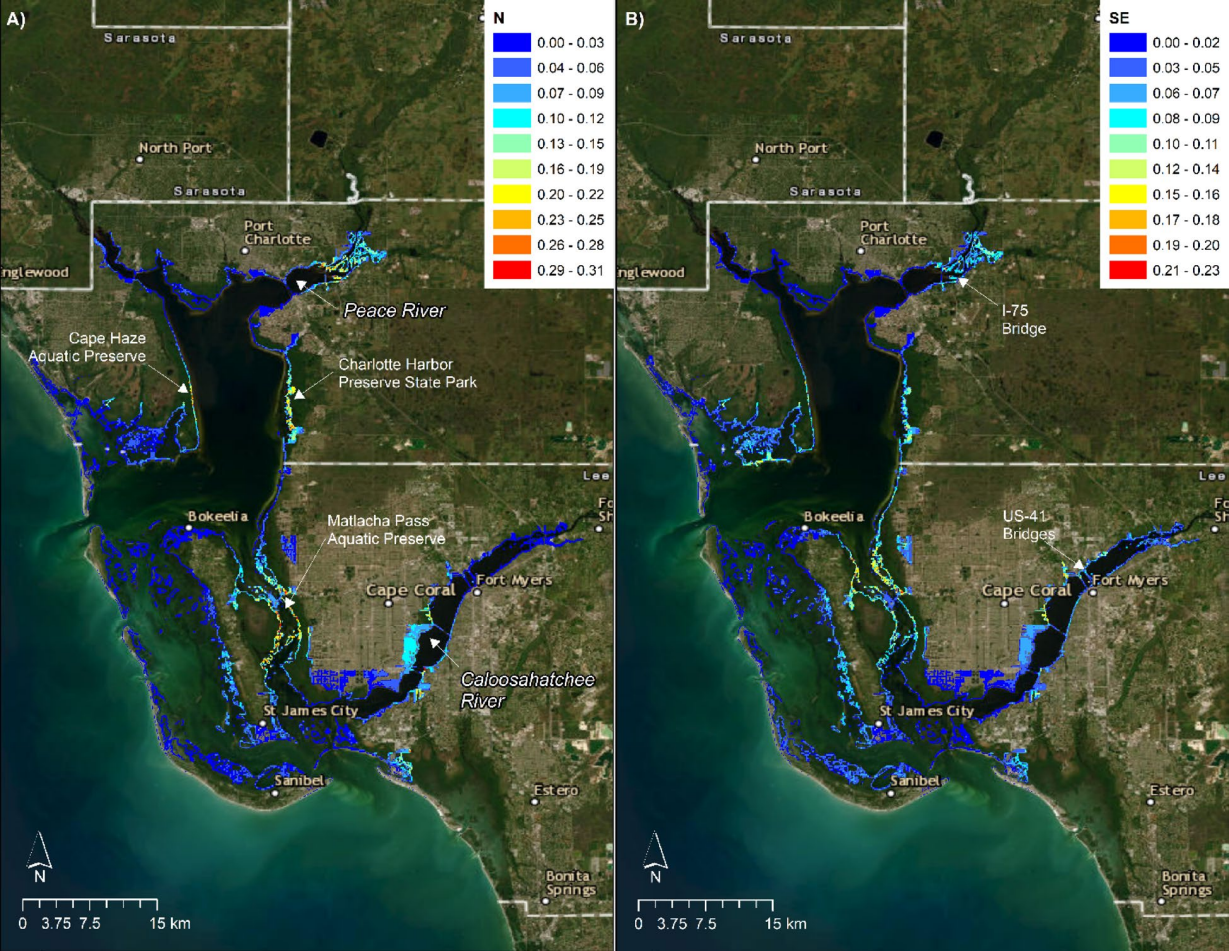
Smalltooth sawfish (*Pristis pectinata*) density in Spring/Summer 2022. Warmer colors denote model-predicted areas of A) relatively high density (N) and B) standard error SE for age-0 and age-1 individuals in Charlotte Harbor under average environmental conditions from March through August 2022, based on interpolation of daily-averaged sonde data by empirical Bayesian kriging. Ranges of SE are included. Basemap used with permission from ESRI World Imagery and its partners.

Density modeling suggested relatively stable patterns of spatial abundance over time. Season was not selected in the best-fitting model, and although estimated abundances varied annually, the centers of abundance were relatively consistent. The model predicted highest abundances in the Peace and Caloosahatchee Rivers in less developed habitats around the I-75 and US-41 bridges, respectively (Fig. 4). Extrapolating beyond the survey domain, the model also predicted high abundance along the eastern shorelines of Charlotte Harbor Preserve State Park and Cape Haze Aquatic Preserve, and within the Matlacha Pass Aquatic Preserve between St. James City and Bokeelia. The spatial distribution was relatively stable across years and seasons, except for relatively higher abundance on the southeastern corner of Pine Island in 2016, apparently driven by lower salinity (Fig. S6).

**Fig. 5:**
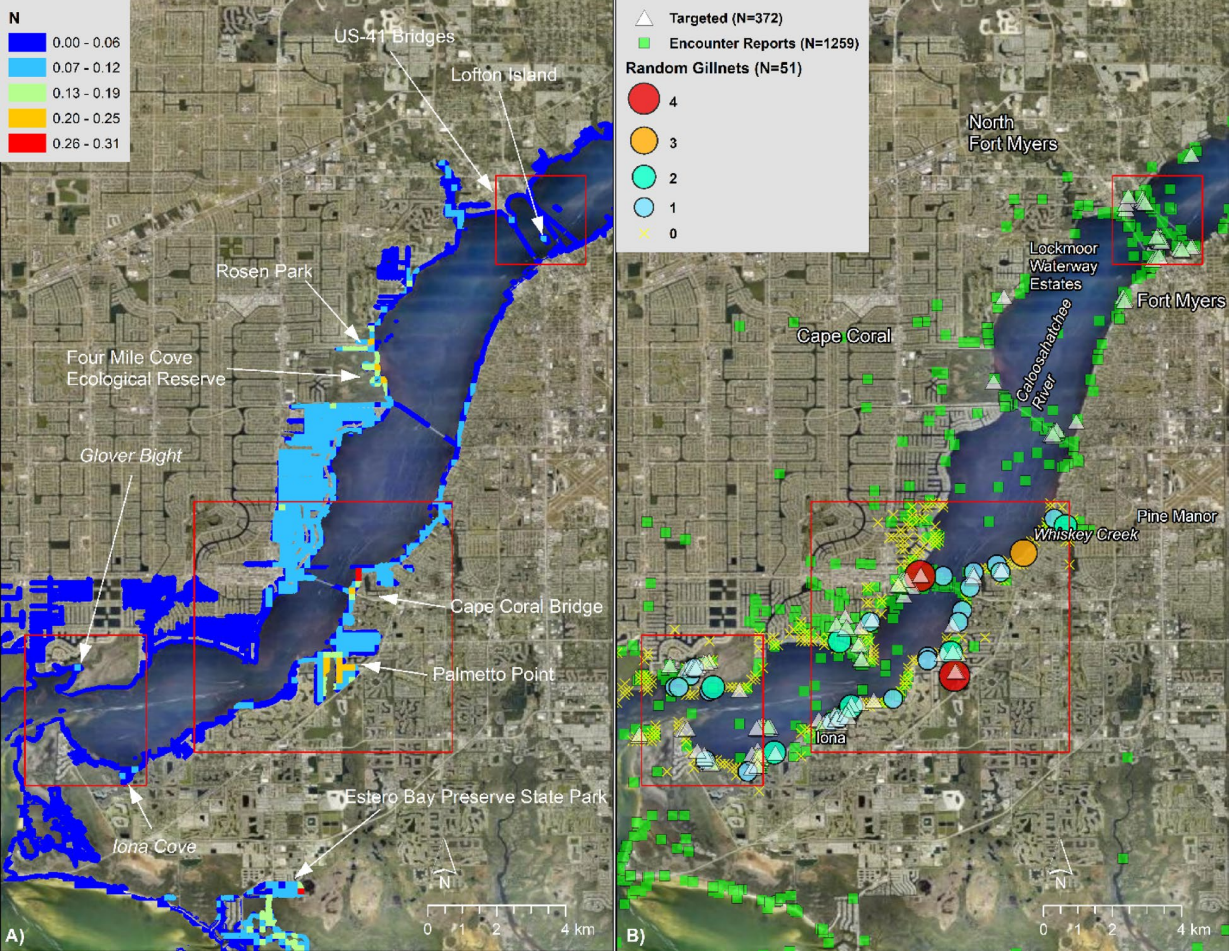
Smalltooth sawfish (*Pristis pectinata*) density in the Caloosahatchee River in Spring/Summer 2021. (**A**) Relative predicted density of age-0 and age-1 individuals for average environmental conditions from March through August 2021, based on interpolation of daily-averaged sonde data by empirical Bayesian kriging; and (**B**) Point data from U.S. Sawfish Recovery Database (2015–2023; all life history stages), targeted sampling (2004–2022; age-0 and age-1 only), and random gillnet sampling (2010–2022). Red polygons denote “high-use areas” or “hotspots” previously identified for small juveniles by [19] and [40]. Basemap used with permission from ESRI World Imagery and its partners.

Our analysis suggested relatively high abundance in the Caloosahatchee River near Glover Bight, Iona Cove, and between the Cape Coral and US-41 bridges, particularly near Palmetto Point, the Four Mile Cove Ecological Preserve, Rosen Park, Lofton Island, the undeveloped spits of mangrove habitat emerging from Lochmoor Waterway Estates and the terminus of Moody River Boulevard (Fig. 5). The model also predicted relatively high abundance in Estero Bay Preserve State Park.

**Fig. 6:**
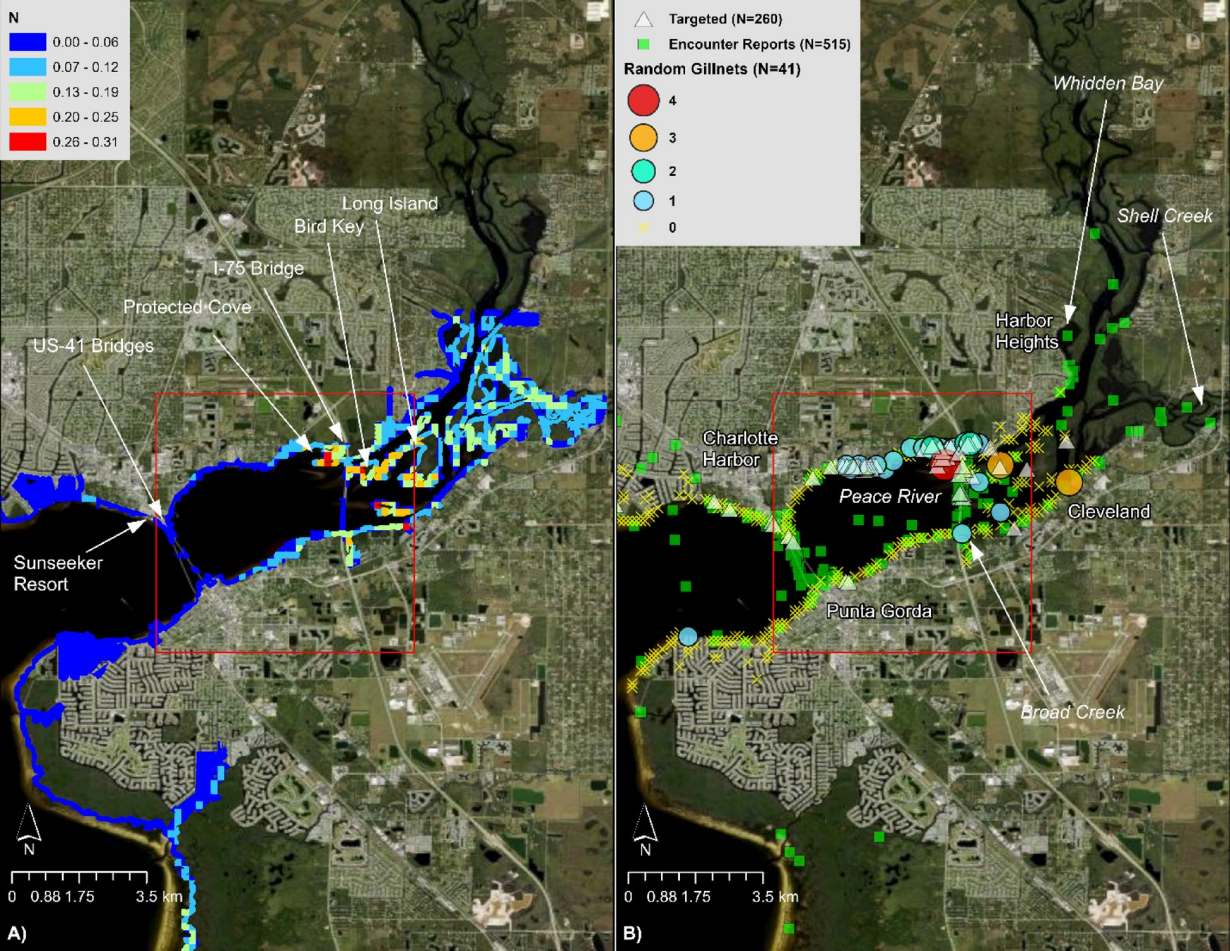
Smalltooth sawfish (*Pristis pectinata*) density in the Peace River in Spring/Summer 2021. (**A**) Relative predicted density of age-0 and age-1 individuals for average environmental conditions from March through August 2021, based on interpolation of daily-averaged sonde data by empirical Bayesian kriging; and (**B**) Point data from U.S. Sawfish Recovery Database (2015–2023; all life history stages), targeted sampling (2004–2022; age-0 and age-1 only), and random gillnet sampling (2010–2022). Red polygons denote the “high-use area” or “hotspot” previously identified for small juveniles by [19] and [40]. Basemap used with permission from ESRI World Imagery and its partners.

Our analysis predicted relatively high abundance in the Peace River surrounding the I-75 bridges, including the protected cove (locally referred to as “the bayou”) referenced in [31] and the natural habitats around Broad Creek and the mid-river islands such as Bird Key and Long Island (Fig. 6). Additionally, the model suggested relatively high abundance in the riverine and creek habitats (i.e., Shell Creek, Peace River to Whidden Bay) between the neighborhoods of Cleveland and Harbor Heights.

### Abundance estimation

**Fig. 7:**
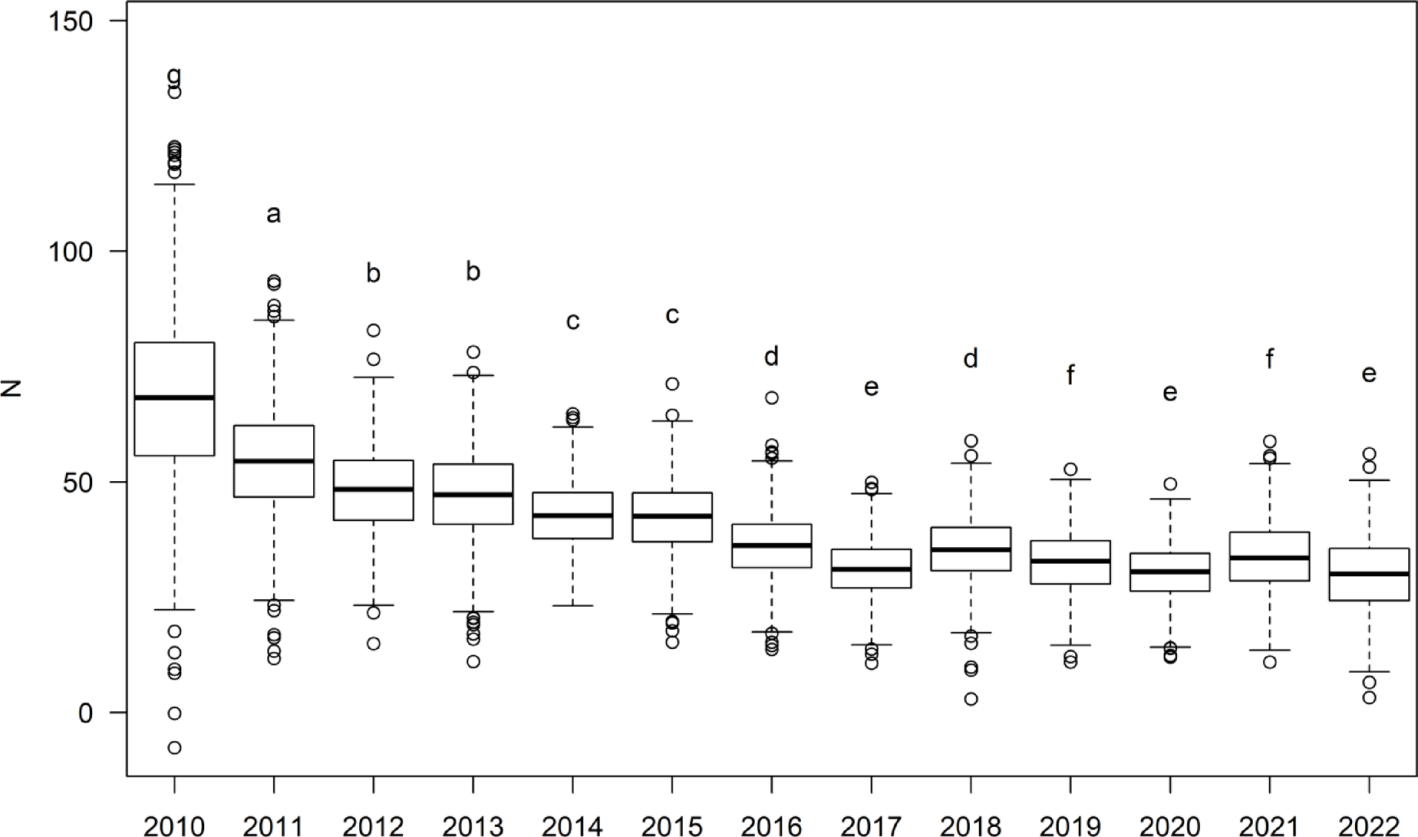
Declining trend in smalltooth sawfish (*Pristis pectinata*) relative abundance. Model-estimated Spring/Summer abundance (N) of age-0 and age-1 individuals in Charlotte Harbor from random gillnet sampling, accounting for parameter uncertainty and environmental variability. Lettering denotes significant differences revealed by post-hoc pairwise comparisons using Tukey tests.

Model estimation suggests Spring/Summer smalltooth sawfish abundance both within the random gillnet sampling area and across the Charlotte Harbor domain dramatically declined between 2010 to 2011, again from 2011 to 2012, and continued a gradual decline through 2015, followed by biennial fluctuations around a lower level of abundance (Fig. 7). The change in model-estimated Spring/Summer small juvenile abundance across the sampled domain, averaged across all years, was 0.94 ± 0.11 (range: 0.75–1.16). From 2010 to 2022, within the sampled domain, mean estimated annual abundance across all seasons was 34 ± 11 individuals (range: 15–69). Spring/Summer abundance was higher than Fall/Winter and estimated at 40 ± 11 individuals (range: 29–69). Across the broader Charlotte Harbor domain, median estimated Spring/Summer abundance was 503 ± 163 individuals (range: 357–1009).

**Fig. 8:**
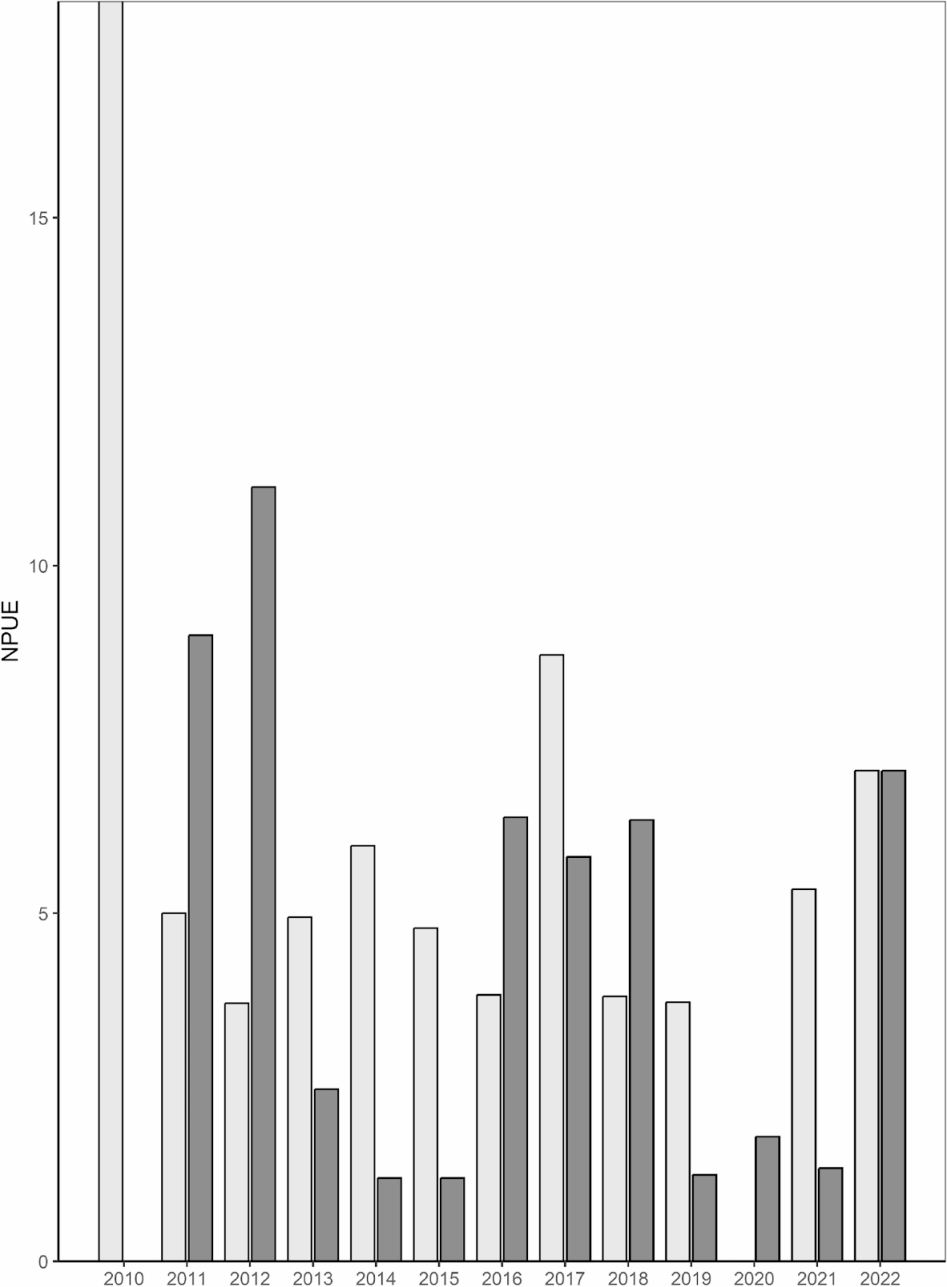
Trends in smalltooth sawfish (*Pristis pectinata*) random gillnet catch by age. Number of age-0 (light gray) and age-1 (dark gray) individuals caught per relative unit effort (NPUE) during 2010–2022.

Raw CPUE data revealed all sawfish captured in 2010 were age-0, whereas age-1 sawfish were also encountered in all subsequent years (Fig. 8). In 2011, 2012, 2016, 2018, and 2020, age-1 individuals comprised most of the catch in random gillnet sampling.

Back-calculating reproductive capacity assuming a uniform distribution for estimated brood sizes of 7–14 individuals and a biennial female reproductive cycle based on Spring/Summer juvenile abundances yielded a brood size estimate of 48 ± 16 (range: 26–144) adult females within the broader Charlotte Harbor domain. Assuming a stable age distribution, we estimated 87 ± 30 (range: 39–211) adult females within the broader Charlotte Harbor domain.

## Discussion

This study provides a comprehensive evaluation of over a decade of small juvenile smalltooth sawfish data from the Charlotte Harbor estuarine system. Our model predicted the highest densities of this endangered species in spatiotemporally stable natural shoreline habitats near concentrations of mangroves, in well-oxygenated (DO > 7 mg/L), warm (25–33 °C), brackish (salinity 5–27) waters between 5 and 15 km upriver in both riverine nurseries. Abundance declined from 2010 through 2017, with some stabilization at low levels since 2018. The predictive utility of our model was confirmed by internal and external validation approaches, along with comparisons to previous electivity and acoustic analyses [19, 31].

Our results, and previous research, show temporal stability in high-density areas (i.e., “hotspots”, “high-use areas” [10]) for age-0 and age-1 individuals [19, 40]). This finding is supported by catch, public encounter, and acoustic data in multiple nurseries, which have found that age-0 (< 1.5 m STL) juveniles reside in and have fidelity to specific high-use areas within the broader nurseries for several months and increase their home range with growth [20, 29, 30, 40]. The association with shallow water (< 0.9 m) weakens for age-1 juveniles, but frequent relocations support strong site fidelity despite larger (1–5 km^2^) home ranges [21, 29, 31]. Parturition site fidelity is common in elasmobranchs [62], but the strong site fidelity to such specific high-use areas is unique to smalltooth sawfish.

In addition to confirming the perennial high-use areas, the model suggested a few other important areas such as Cape Haze Aquatic Preserve, Matlacha Pass Aquatic Preserve, and Charlotte Harbor Preserve State Park. External validation (i.e., targeted sampling and encounter reports) confirmed some small juvenile use of Cape Haze and Matlacha Aquatic Preserves (see Fig. 1E). In contrast, and despite the presence of what appears to be high quality habitat, Charlotte Harbor Preserve State Park is not currently supporting small juveniles. Philopatry [28], site fidelity to specific high-use areas, and limited juvenile home range likely explain much of the patchy distribution of small juveniles, despite seemingly similar habitats elsewhere. Other considerations that may influence habitat use during early life history could include whether adult females follow deep channels through the estuary to their preferred birth sites (i.e., near high-use areas, [29, 30]), or yet to be identified environmental cues (e.g., natal imprinting) that consistently draw gravid females to specific birthing sites.

The parturition site fidelity of smalltooth sawfish is further emphasized by the stability of predicted high-use areas within the broader nurseries across years and seasons in Charlotte Harbor (see Fig. S7) and Everglades/Ten Thousand Islands [40, 63]. This temporal stability shows that focusing conservation efforts on these areas would be an effective recovery strategy and provide a high conservation benefit [31]. Destruction of habitats in these high-use areas should be prohibited and their long-term preservation should be prioritized to prevent extinction and promote recovery.

Elasticity values (i.e., the proportional sensitivities of the finite rate of increase to small changes in matrix elements of survivorship and reproduction) from population viability analysis suggest increased survivorship of juveniles would have the largest relative impact on population recovery (see Fig. 1 in [59]). The high-use areas in Charlotte Harbor contain shallow, red mangrove-lined shorelines that provide juveniles refuge from predators and abundant food sources. Similar modeling efforts in Everglades/Ten Thousand Islands by [63] revealed strong correlations between the presence of small juveniles and environmental variables, with preferential selection of shallow, warm, saline waters fringed with mangroves. The dorso-ventrally compressed bodies of sawfish allow them to use shallow waters (< 0.9 m) that are inaccessible by predators such as large sharks, while mangrove prop roots provide additional refuge during higher tidal stages. Although they are designated as juvenile critical habitat [26] and afforded protection under the ESA, the undeveloped shorelines that characterize many of these high-use areas in the Charlotte Harbor estuarine system are increasingly rare and under continual threat of destruction by human activities. As such, permanent protection of these unique areas is essential to sustain and rebuild the smalltooth sawfish population.

Our model appeared to overestimate the relative importance of canals with hardened seawalls on the Cape Coral side of the Caloosahatchee River between the Cape Coral and Midpoint bridges. Most catches have occurred along main stem river shorelines of these developed neighborhoods as opposed to canals within them (see Fig. 5B). Similarly, previous studies have reported small juveniles near red mangrove habitat 10 times more often than at seawalls [64]. Although the model indicated a strong preference for natural vs. developed shorelines, it appears to overvalue the physicochemical characteristics of this neighborhood. Future efforts to include terms for prey and predator distribution, bottom type, or swimming distance to riverine foraging habitats might improve overall model fits and reduce potentially spurious predictions along developed shorelines or in dredged canals where refuge from predation and prey supply may both be limited. Although expansion of high-use areas through restoration activities that convert nearby hardened seawalls into living shorelines could improve foraging and shelter opportunities for juvenile sawfish [65], protection of existing high-use areas should be prioritized.

Our model indicates a significant decline in age-0 and age-1 smalltooth sawfish abundance in Charlotte Harbor since 2010, with lows in recent years (see Fig. 7). This decline is discouraging given regulatory actions expected to benefit the species such as the state gillnet ban in 1994, the ESA listing of the species in 2003, and the juvenile critical habitat designation in 2009. Despite these protections, coastal development in Charlotte Harbor has proceeded at an aggressive pace, with associated losses of mangrove habitats, increased vessel traffic, and negative impacts to water quality [66, 67]. Mangrove habitats have declined in the Charlotte Harbor region because of permitted development, hurricane damage [68], and sea level rise [69]. Long-term water quality trends in the estuarine system suggest declining dissolved oxygen, increased fecal coliform, and increased nitrogen [70]. Similarly, Lake Okeechobee freshwater releases into the Caloosahatchee River lead to abrupt shifts in salinity, dissolved oxygen, and nutrient loading [71]. Effects of these trends on the fitness and behavioral decisions of adult females and small juveniles is unknown.

In addition to major consistent anthropogenic mortalities from fisheries such as the U.S. southeast shrimp trawl fishery [35, 36], smalltooth sawfish are also killed by stochastic natural events. For example, the average 12-day temperature of 9.3 °C between 2 and 13 January 2010 was the lowest on record for any 12-day period in Naples, Florida, since 1981 [72], resulting in widespread fish kills including several reports of dead juvenile sawfish in Charlotte Harbor [46]. This kill took place prior to sampling in 2010 and appears to have reduced the number of age-1 sawfish in the estuary but did not appear to affect the incoming 2010 young-of-year, which were likely born after the cold shock event (see Fig. 8). This may partly explain the precipitous drop in abundance from 2010 to 2011, but would not account for the continued decline (see Fig. 7).

In 2024 and 2025, there were also unprecedented mortality events affecting large juvenile and adult (> 3 m STL) smalltooth sawfish. From January to August 2024, at least 230 individuals demonstrated unusual swimming behavior in shallow water (e.g., circling, thrashing). At least 56 of these individuals were confirmed dead. The specific cause of the event and its effects on the species remains under investigation but could be a water-borne toxin or combination of toxins from benthic algae (A. Robertson, University of South Alabama, pers. comm.). There is a high probability that most affected individuals perished, considering the only rescued individual did not survive, despite intensive veterinary care. Given that smalltooth sawfish are demersal and may not float when they die, it is also likely that many mortalities went unobserved. A similar mortality event occurred in 2025; as of 26 June 2025, 68 affected sawfish and 9 confirmed mortalities had been reported. These events were centered in the Florida Keys; however, unusual swimming behaviors and associated mortalities were reported as far north as Bay Pines (27.8092° N) and St. George Island, Florida (29.6585° N). Given the substantial adult connectivity between the Florida Keys and Charlotte Harbor [23], our analysis provides a useful baseline to evaluate the impacts of these concerning mortality events, which may become apparent in future recruitment patterns and assessments of the genetic diversity of the population.

We back-calculated adult female abundance based on brood size (48 ± 16) and stable age distribution (87 ± 30). The brood size approach likely produced a lower estimate, given it does not consider natural mortality on age-0 and age-1 juveniles. Based on reconstruction of parental genotypes from the study area, [28] estimated that 55 females gave birth to 142 broods over a 12-year period (2003–2015). This estimate would likely be lower than the estimate generated, assuming a stable age distribution across the broader Charlotte Harbor domain, as genetic samples were collected mostly from the two riverine nurseries. Regardless, the convergence of these three independent approaches (i.e., 48, 55, or 87 females) is strong evidence that fewer than 100 adult females are supporting the smalltooth sawfish population in Charlotte Harbor. Parental reconstruction from samples taken in the larger Everglades/Ten Thousand Islands nursery habitat yielded only 71 female genotypes [73]. The low number of reproductive females and high quasi-extinction risk for this species [59], particularly when considering current natural and anthropogenic sources of mortality [36], suggests a species at risk of extinction with urgent need of management intervention.

In the U.S., NMFS is charged with promoting recovery of the smalltooth sawfish. Under Sect. 7 of the ESA, agencies must consult with NMFS to ensure their proposed actions (e.g., coastal development, fishery bycatch) do not jeopardize the survival of listed species. Understanding the distribution, abundance, migration patterns, and site fidelity of sawfish is essential for accurately estimating the impact of proposed activities, especially within Charlotte Harbor nurseries. Nurseries within the Everglades/Ten Thousand Islands critical habitat area are rarely affected by development projects due to restrictions associated with Everglades National Park. Anthropogenic impacts to individuals can be estimated as the product of: (1) the probability of an activity occurring in an area; (2) the probability of an individual being in an activity area, often expressed as a distribution model; (3) the duration of exposure of the individual to the activity, often measured as site fidelity; and (4) the probability of the activity affecting the individual, often expressed as a dose–response curve [74]. Managers can work with action agencies to schedule activities when risk is minimized and to enact conservation measures to reduce the level and duration of exposure. This may be particularly effective when considering spatiotemporal closures to fishing or environmental windows for actions with temporary in-water work that does not permanently alter the habitat. Even when duration of exposure and probability of adverse effects are unknown, relative risk assessments can be used to identify preferred alternatives [6–8]. Our results and findings from acoustic telemetry studies [20, 21, 29, 30, 40] could be used to predict both the number of individuals exposed and duration of exposure to planned activities such as shoreline construction projects.

In conclusion, immediate, robust recovery actions are necessary to prevent the smalltooth sawfish from going extinct. The U.S. population is the last remaining stronghold for the species in its entire range. Minimizing known, preventable threats is critical to recovery. Examples of actionable activities that would directly benefit the species include: 1) develop management strategies to reduce bycatch in the southeast shrimp trawl fishery; 2) purchase and protect known high-use portions of nurseries from future anthropogenic perturbations in perpetuity through conservation easements, state acquisitions, or establishment of an in-lieu fee program (e.g., [75]); and 3) replace ball bungee cords currently used to secure boat canopy covers with another mechanism and encourage their targeted removal before storms introduce them into waterways [34]. Finally, consistent, long-term funding is needed to stabilize and maintain research, monitoring, and outreach efforts to support the conservation and management needs of the species. Future studies should focus on filling gaps in our understanding of smalltooth sawfish biology and ecology by establishing a similar baseline for small juveniles in the Everglades/Ten Thousand Islands region, prioritizing estimates of population abundance (e.g., effective and census population sizes), and providing opportunities for genetic sampling to inform close-kin mark-recapture genetic population estimates [76].

## Supplementary Information

Below is the link to the electronic supplementary material.

Supplementary Material 1

## Data Availability

The datasets generated and/or analyzed during the current study are not publicly available due to the smalltooth sawfish being an endangered species, but are available from the corresponding author on reasonable request. External environmental parameters used in the model are publicly available as listed in Table 1 and in references. The R and Python code for data processing are available from the corresponding author upon request.
