# Correction: Long-term random sampling confirms high-use areas and indicates declining abundance of juvenile smalltooth sawfish (*Pristis pectinata*) in Charlotte Harbor, Florida

**DOI:** 10.1038/s41598-026-48537-9

**Published:** 2026-04-29

**Authors:** Nicholas A. Farmer, Adam B. Brame, Rabiya Dar, Andrew K. Wooley, Lukas B. Heath, Dylan M. Yakich, Steven M. Lombardo, Gregg R. Poulakis

**Affiliations:** 1 NOAA/National Marine Fisheries Service, Southeast Regional Office, 263 13th Avenue South, St. Petersburg, FL 33701 USA; 2 NOAA/National Centers for Coastal Ocean Science, 1305 East West Highway, Silver Spring, MD 20910 USA; 3 Charlotte Harbor Field Laboratory, Fish and Wildlife Research Institute, Florida Fish and Wildlife Conservation Commission, 585 Prineville Street, Port Charlotte, FL 33954 USA; 4 Bonefish and Tarpon Trust, 2937 SW 27th Avenue, Suite 203, Miami, FL 33133 USA

Correction to: *Scientific Reports* 10.1038/s41598-025-14430-0, published online 10 March 2026

The original version of this Article contained an error, in the *Methods* section of the main text, which occurred during the production process. Specifically in *Relative abundance estimation* subsection the fourth paragraph that read:

“In this approach, a truncated normal distribution (μ = 157.1322, σ = 111.5191, a = 54.7485, b = 569.8908) was used to generate 1000 realizations of age-0 and age-1 females and a uniform distribution (a = 7, b = 11) was used to set the lower bound for maturity. Ratios from the stable age distribution were then used to generate 1000 realizations of the resultant number of adult females. In both approaches, the resultant expected number of adult females contributing to offspring in Charlotte Harbor was compared to the estimated number of adult females from parental genotype reconstruction by [28].”

Now reads:

“In this approach, a truncated normal distribution (μ = 251.481, σ = 81.41511, a = 178.511, b = 504.7369) was used to generate 1000 realizations of age-0 and age-1 females and a uniform distribution (a = 7, b = 11) was used to set the lower bound for maturity. Ratios from the stable age distribution were then used to generate 1000 realizations of the resultant number of adult females. In both approaches, the resultant expected number of adult females contributing to offspring in Charlotte Harbor was compared to the estimated number of adult females from parental genotype reconstruction by [28].”

The original Article has been corrected.

