# Supplementary material for: Long-term random sampling confirms high-use areas and indicates declining abundance of juvenile smalltooth sawfish ( Pristis pectinata ) in Charlotte Harbor, Florida
Source: Sci Rep. 2026 Mar 10;16:8736. doi: 10.1038/s41598-025-14430-0 (PMC12979683; doi:10.1038/s41598-025-14430-0)
Supplement: Supplementary file 1 — Supplementary Material 1 [file 41598_2025_14430_MOESM1_ESM.docx]

# **Supplemental Figures**


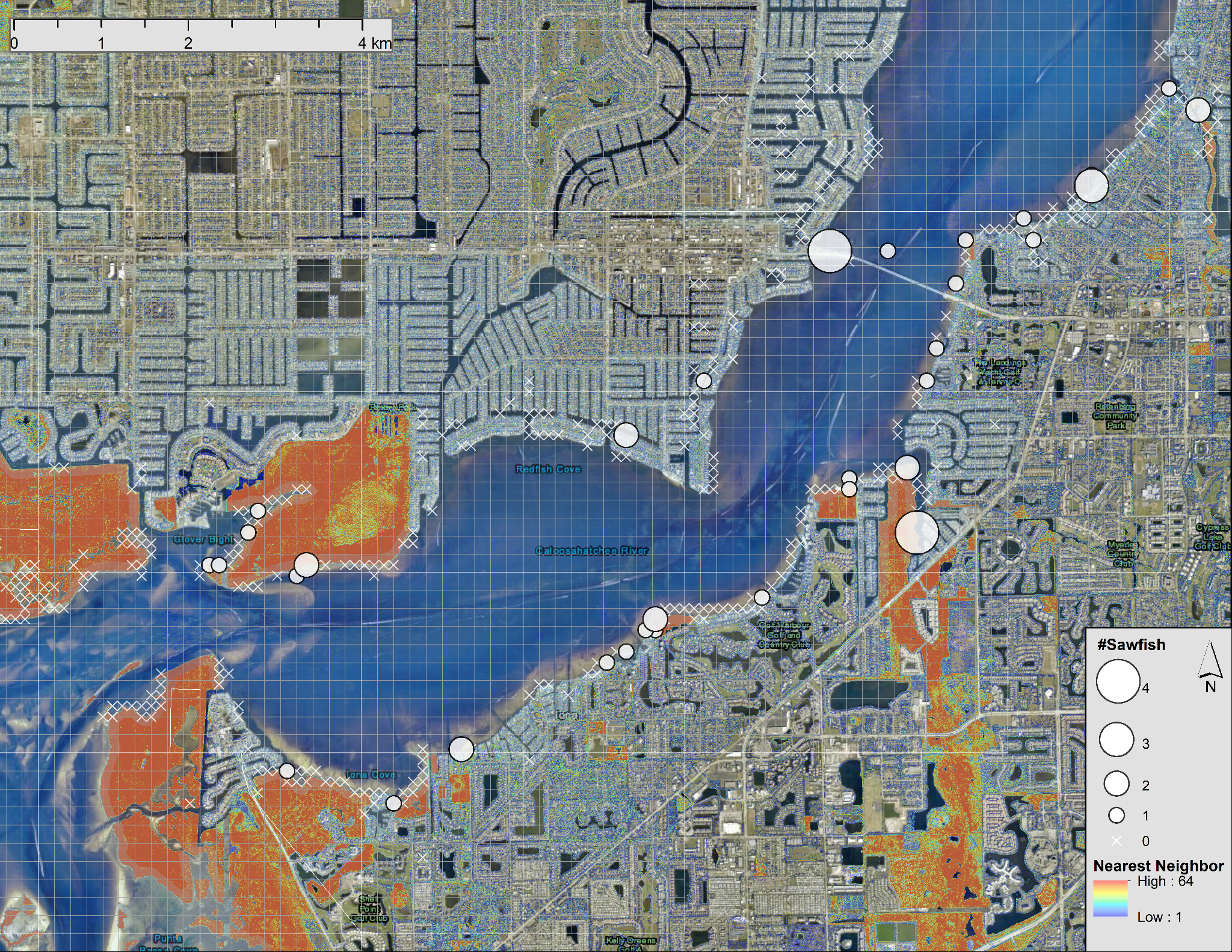


**Fig. S1.** *Caloosahatchee River gillnet sampling.* Random gillnet sampling (Xs) and catches (circles) of age-0 and age-1 smalltooth sawfish in the Caloosahatchee River, relative to high concentrations of mangrove habitat identified from classification of aerial imagery. Basemap used with permission from ESRI World Imagery and its partners.


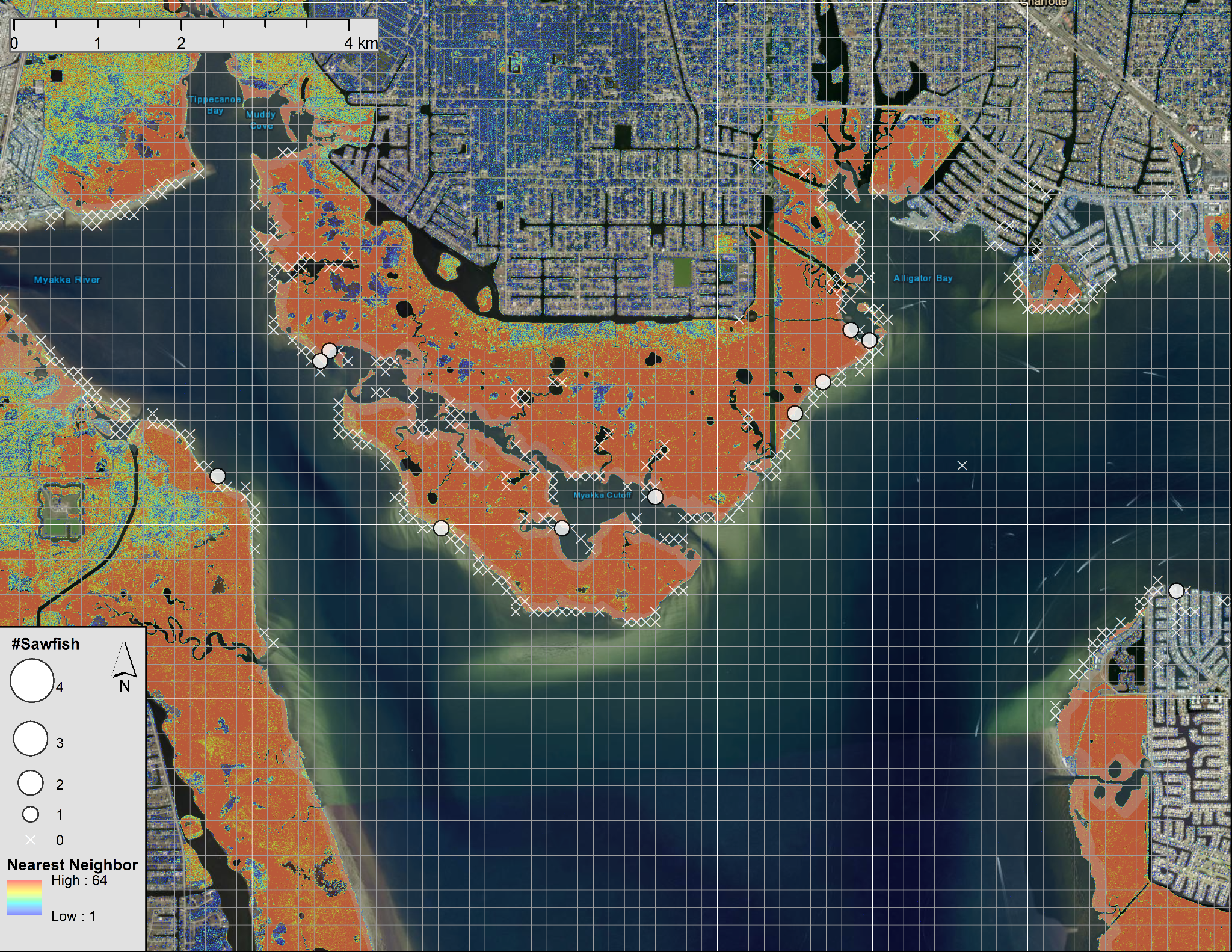


**Fig. S2.** *Myakka and Peace River gillnet sampling.* Random gillnet sampling (Xs) and catches (circles) of age-0 and age-1 smalltooth sawfish near the mouths of the Myakka (left) and Peace (right) rivers, relative to high concentrations of mangrove habitat identified from classification of aerial imagery. Basemap used with permission from ESRI World Imagery and its partners.


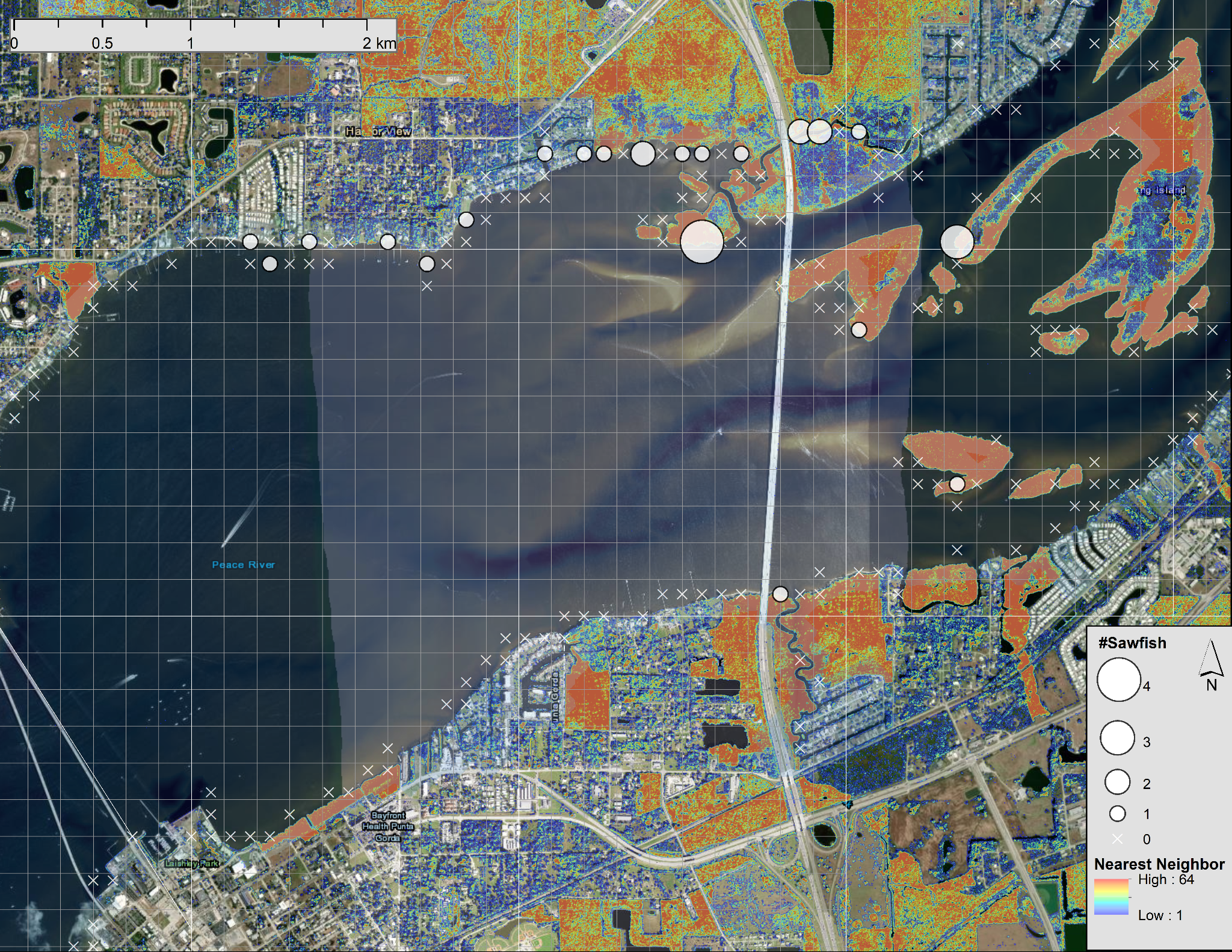


**Fig. S3.** *Peace River gillnet sampling.* Random gillnet sampling (Xs) and catches (circles) of age-0 and age-1 smalltooth sawfish in the Peace River, relative to high concentrations of mangrove habitat identified from classification of aerial imagery. Basemap used with permission from ESRI World Imagery and its partners.


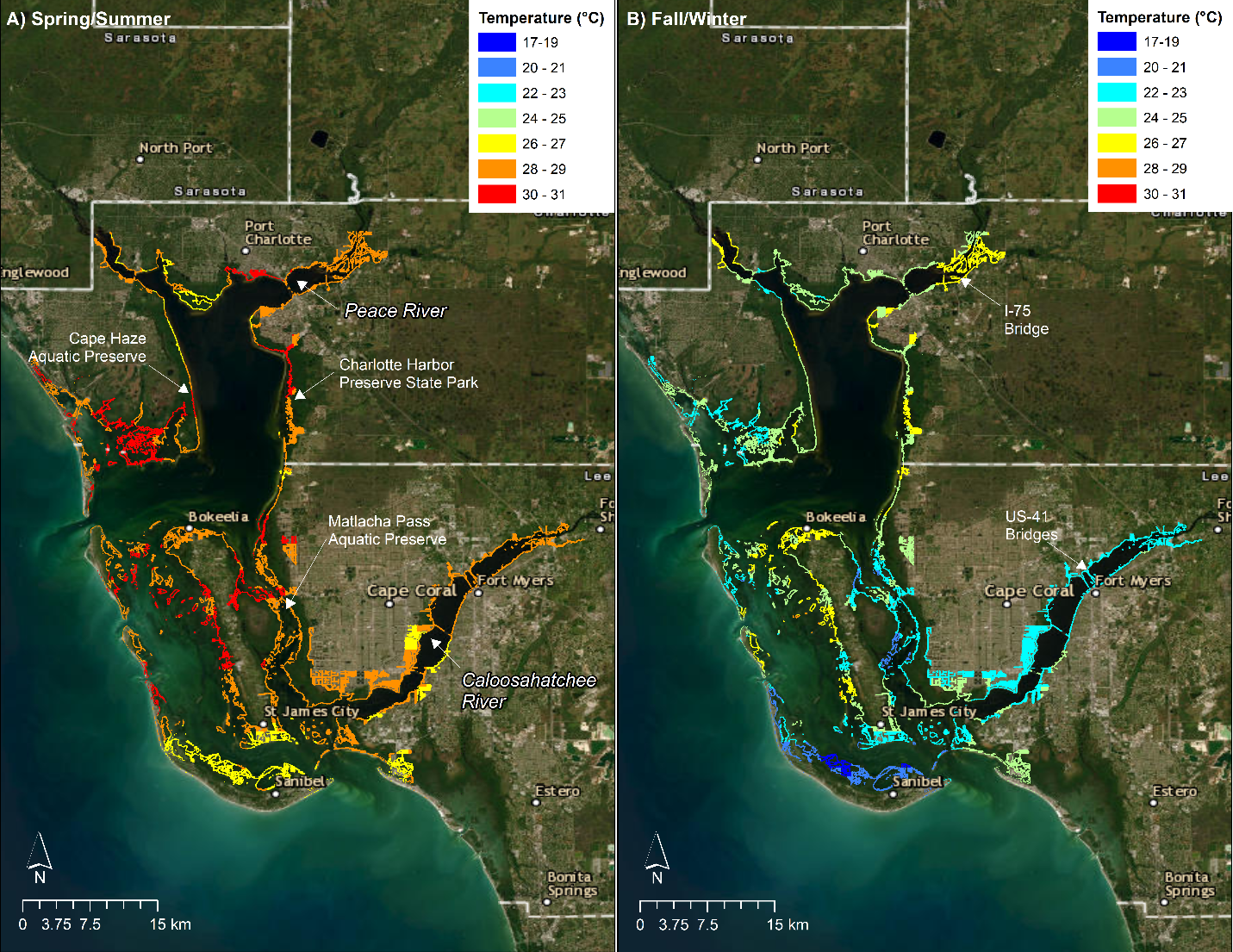


**Fig. S4.** *Interpolated temperature 2022.* Empirical Bayesian kriging output of interpolated temperatures from daily averaged sonde data for the Charlotte Harbor domain in A) Spring/Summer and B) Fall/Winter 2022. Basemap used with permission from ESRI World Imagery and its partners.


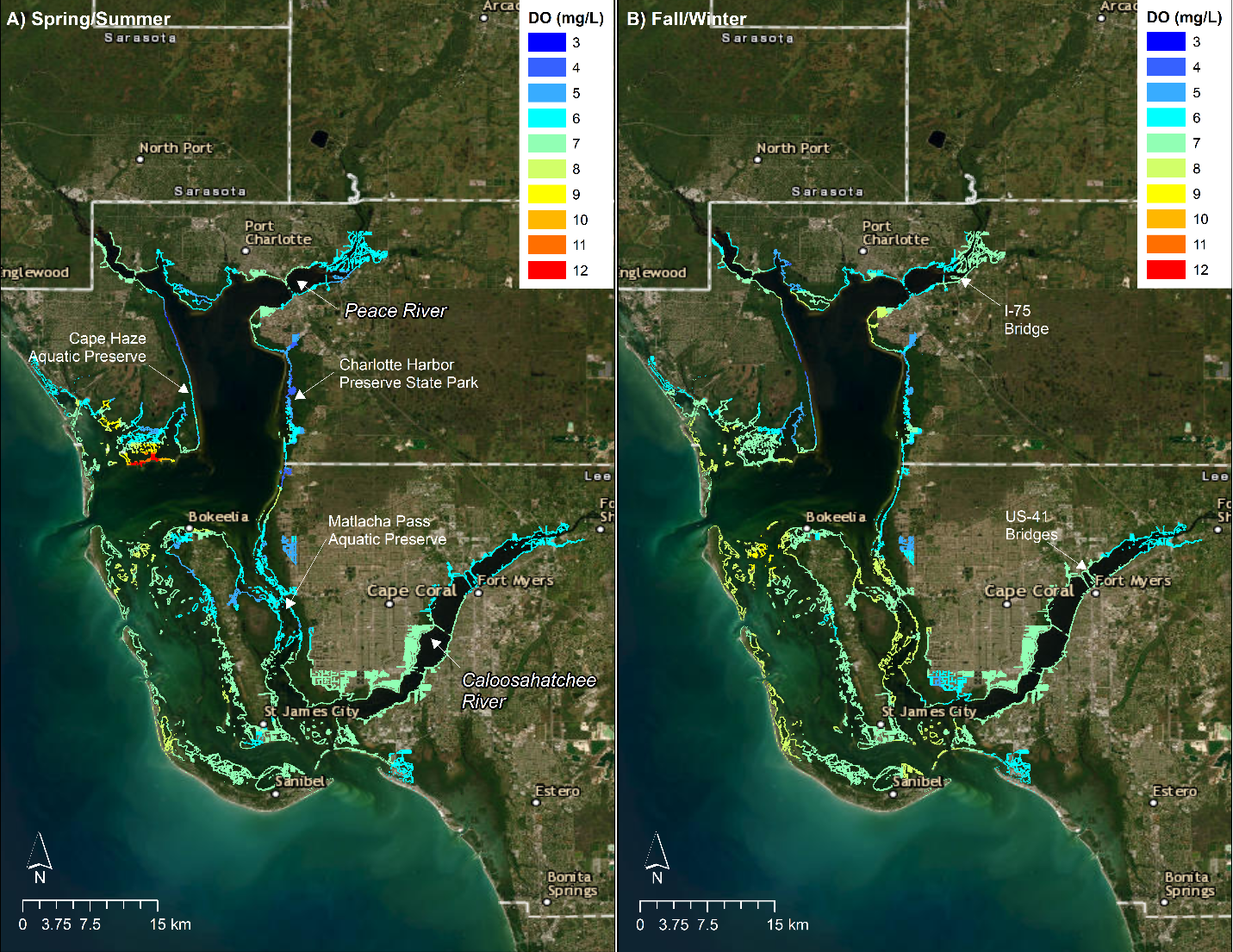


**Fig. S5.** *Interpolated dissolved oxygen 2022.* Empirical Bayesian kriging output of interpolated dissolved oxygen (DO) from daily averaged sonde data for the Charlotte Harbor domain in A) Spring/Summer and B) Fall/Winter 2022. Basemap used with permission from ESRI World Imagery and its partners.


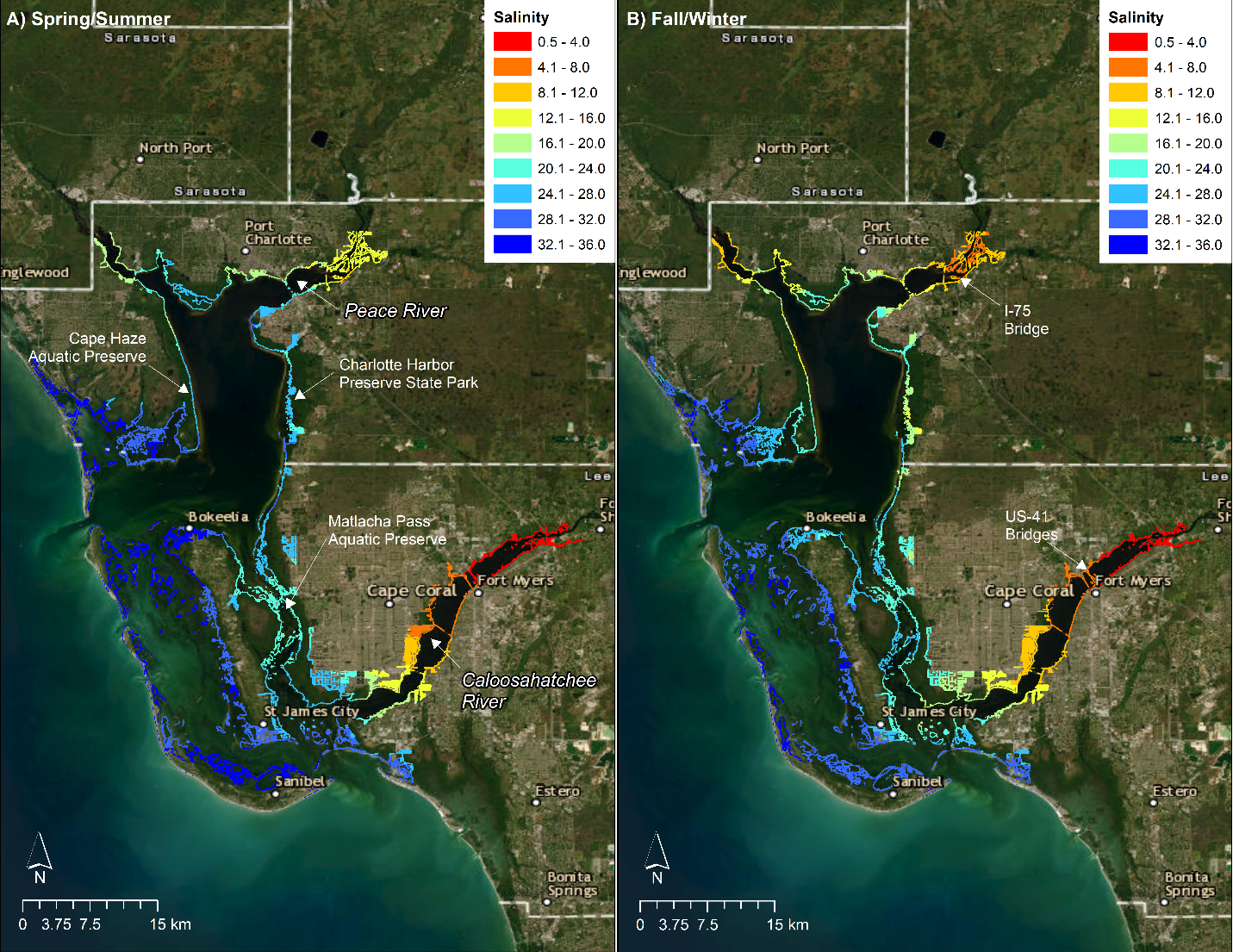


**Fig. S6.** *Interpolated salinity 2022.* Empirical Bayesian kriging output of interpolated salinity from daily averaged sonde data for the Charlotte Harbor domain in A) Spring/Summer and B) Fall/Winter 2022. Basemap used with permission from ESRI World Imagery and its partners.


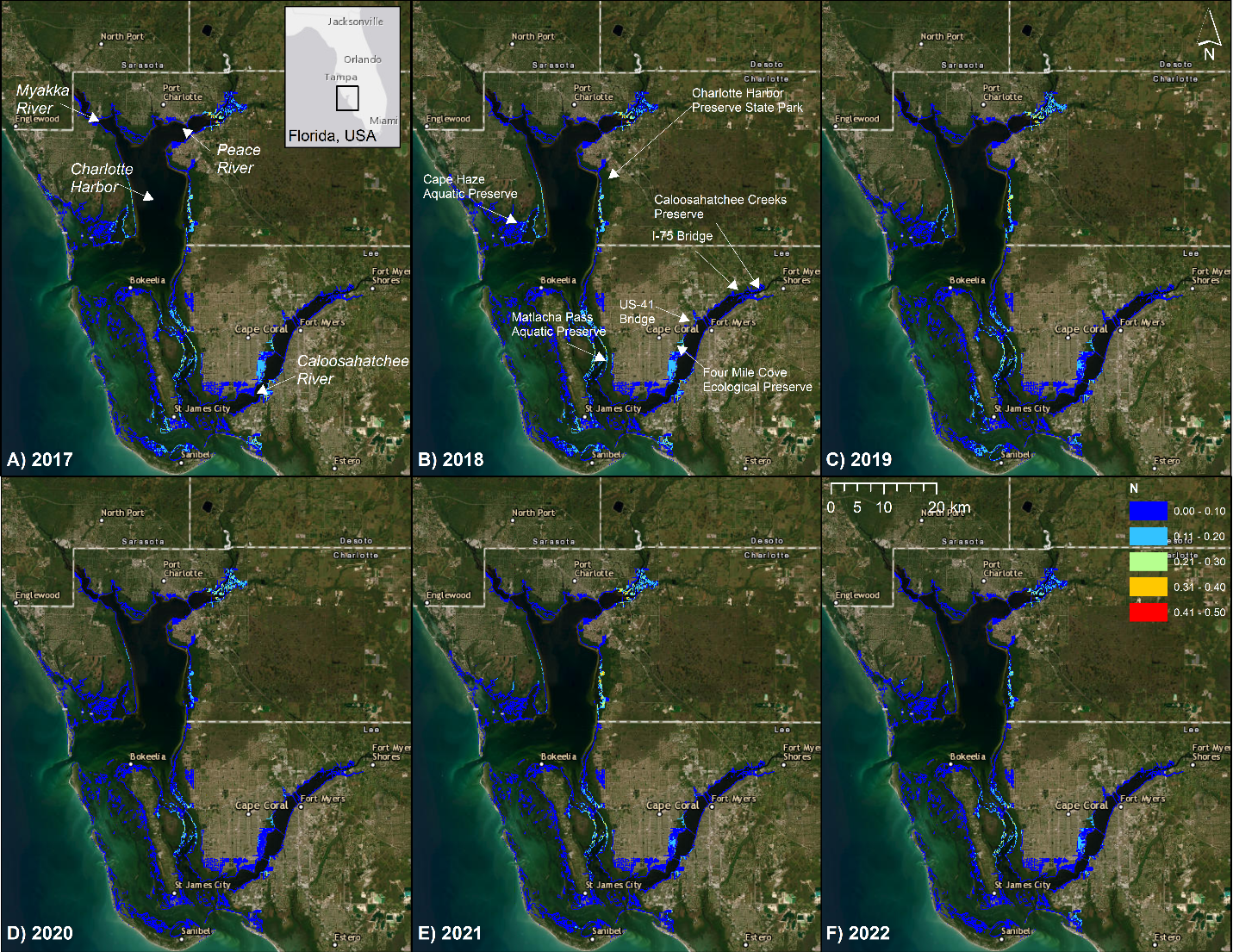


**Fig. S7.** *Smalltooth sawfish density 2017–2022*. Predicted density of age-0 and age-1 smalltooth sawfish for average environmental conditions in Spring/Summer, based on model fits to daily-averaged sonde data interpolated by empirical Bayesian kriging. Warmer colors denote relatively higher predicted relative abundance. Basemap used with permission from ESRI World Imagery and its partners.
